# Targeting the Fibroblast Growth Factor Receptor (FGFR) Family in Lung Cancer

**DOI:** 10.3390/cells10051154

**Published:** 2021-05-10

**Authors:** Laura Pacini, Andrew D. Jenks, Nadia Carvalho Lima, Paul H. Huang

**Affiliations:** Division of Molecular Pathology, The Institute of Cancer Research, London SM2 5NG, UK; Laura.Pacini@icr.ac.uk (L.P.); Andrew.Jenks@icr.ac.uk (A.D.J.); nadia.sofia.c.l@hotmail.com (N.C.L.)

**Keywords:** FGFR, lung cancer, tyrosine kinase inhibitors, drug resistance

## Abstract

Lung cancer is the most common cause of cancer-related deaths globally. Genetic alterations, such as amplifications, mutations and translocations in the fibroblast growth factor receptor (FGFR) family have been found in non-small cell lung cancer (NSCLC) where they have a role in cancer initiation and progression. *FGFR* aberrations have also been identified as key compensatory bypass mechanisms of resistance to targeted therapy against mutant epidermal growth factor receptor (EGFR) and mutant Kirsten rat sarcoma 2 viral oncogene homolog (KRAS) in lung cancer. Targeting FGFR is, therefore, of clinical relevance for this cancer type, and several selective and nonselective FGFR inhibitors have been developed in recent years. Despite promising preclinical data, clinical trials have largely shown low efficacy of these agents in lung cancer patients with *FGFR* alterations. Preclinical studies have highlighted the emergence of multiple intrinsic and acquired resistance mechanisms to FGFR tyrosine kinase inhibitors, which include on-target *FGFR* gatekeeper mutations and activation of bypass signalling pathways and alternative receptor tyrosine kinases. Here, we review the landscape of FGFR aberrations in lung cancer and the array of targeted therapies under clinical evaluation. We also discuss the current understanding of the mechanisms of resistance to FGFR-targeting compounds and therapeutic strategies to circumvent resistance. Finally, we highlight our perspectives on the development of new biomarkers for stratification and prediction of FGFR inhibitor response to enable personalisation of treatment in patients with lung cancer.

## 1. Introduction

The Fibroblast Growth Factor Receptor (FGFR) family plays a central role in a broad range of important physiological events during embryonic development and adult response to injury, tissue repair and regeneration [1,2,3]. FGFRs are key to the regulation of a number of cellular processes such as survival, proliferation, migration, differentiation and metabolism [4,5,6,7]. They are also involved in the development and progression of several cancer types, including lung cancer. Here we review the landscape of FGFR aberrations inherent in lung cancer or found in patients that progress on targeted therapy treatment as a compensatory bypass pathway. We also discuss the preclinical and clinical advances in targeting these aberrations, as well as the acquired resistance mechanisms to FGFR inhibitors and therapeutic approaches to overcome drug resistance.

### 1.1. An Overview of the FGFR Family 

**Figure 1:**
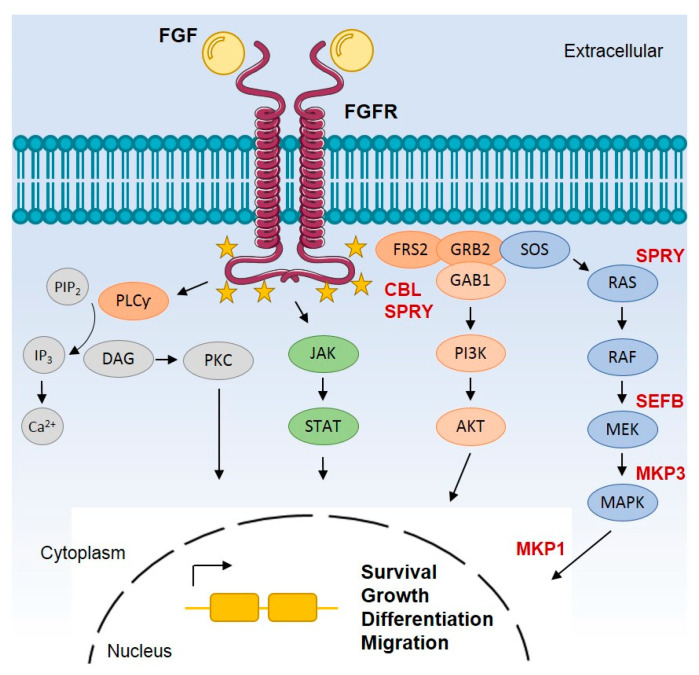
Simplified overview of FGFR canonical signalling pathways. FGFs bind to FGFR inducing receptor dimerisation which then drives the transphosphorylation of the tyrosine kinase domain in the intracellular compartment of the cell. The intracellular portion of active FGFR is phosphorylated at multiple tyrosine sites. In the C-terminus, tyrosine phosphorylation acts as a docking site for molecules containing SH2 domains such as PLCγ. Phosphorylation of PLCγ hydrolyses PIP2 to produce DAG and IP3, inducing the release of calcium cations and subsequent activation of PKC. In the intracellular juxtamembrane region of FGFR, phosphorylation leads to the recruitment of FRS2 which then acts as a secondary docking protein to form two independent complexes. One complex is FRS2-GRB2-SOS that activates RAS, which in turn activates the MAPK pathway. A second complex is FRS2-GRB2-GAB1, which drives the activation of PI3K/AKT pathway. Other pathways are also known to be activated by FGFR such as STAT, p38 MAPK, JNK, SRC and RSK2 pathways. Collectively, these pathways play multiple roles in cell survival, growth, migration, differentiation and metabolism. FGFR signalling is regulated by receptor internalisation upon ubiquitination by CBL or by negative modulation by different proteins (shown in bold red) such as MKP, SEF and SPRY. The yellow stars represent tyrosine phosphorylation sites. HS: heparin sulphate; FRS2: FGFR substrate 2; PLCγ: protein phospholipase Cγ; PIP2: phosphatidylinositol 4,5-bisphosphate; DAG: diacylglycerol; IP3: IP3 inositol 1,4,5-triphosphate; Ca^2+^: calcium; PKC: protein kinase C; GRB2: growth factor receptor-bond 2; SOS: son of sevenless; MAPK: mitogen-activated protein kinase; PI3K: phosphoinositide 3-kinase; MEK: intracellular mitogen-activated protein kinase/Erk kinase; RAF: Raf-1 proto-oncogene, serine/threonine kinase; RAS: proto-oncogene GTPase; GAB1: GRB2-associated binding protein 1; AKT: Akt serine/threonine kinase 1; JAK: janus kinase; STAT: signal transducer and activator of transcription; CBL: Cbl proto-oncogene E3 ubiquitin protein ligase; MKP: MAPK phosphatases; SEF: similar expression to fgf genes; SPRY: sprouty homolog.

The FGFR family is composed of four highly conserved receptor tyrosine kinases (RTKs), FGFR1-4, as well as a fifth member known as FGFR-like protein (FGFR5). FGFR1-4 are transmembrane proteins activated by the binding of a variety of fibroblast growth factor (FGF) ligands. While FGFR1-4 are active kinases, FGFR5 which also localises to the cell membrane and binds FGF ligands, lacks the kinase domain and does not possess kinase activity [8]. There are 22 FGF ligands in mammals that range in size from 150–300 amino acids, and crystallography studies have shown that there is a homologous core domain in all FGFs composed of around 125 amino acids. The region outside the conserved core is comprised of variable amino acid sequences that determine the selectivity of binding of distinct FGFs to different FGFR family members [9]. FGFs bind to FGFR, resulting in receptor dimerisation which drives the transphosphorylation of the intracellular tyrosine kinase domain inducing the recruitment of adaptor proteins responsible for the activation of several downstream signalling pathways through which these receptors exert their biological functions (Figure 1) [10]. Examples of the most common signalling pathways activated by FGFRs are the rat sarcoma kinase (RAS) and mitogen-activated protein kinase (MAPK), the phosphatidylinositol 3-kinase/protein kinase B (PI3K/AKT), signal transduction and activation of transcription (STAT), the c-Jun N-terminal kinase (JNK) and SRC pathways [4,5,6,7].

### 1.2. The Landscape of FGFR Alterations in Lung Cancer 

Aberrations in FGFR have been implicated in the initiation and progression of several cancer types [11]. Reported FGFR alterations in cancer include receptor translocations, amplifications and point mutations [12]. In addition to these mechanisms, a switch in FGFR splicing isoform, alterations in FGFR internalisation, impaired signalling termination and defective FGF ligand secretion have also been reported to affect FGFR canonical pathways leading to oncogenesis [13,14]. A study from 2015 found through next-generation sequencing (NGS) of almost 5000 tumours across all cancer types that FGFR aberrations (gene amplifications, mutations and rearrangements) are present in 7.1% of these tumours, of which ~13% have been found in lung cancer [15].

Lung cancer is the leading cause of cancer-related death worldwide [16]. It is classified into two main histological subtypes: non-small cell lung cancer (NSCLC) and small cell lung cancer (SCLC), which account for ~85% and ~15% of cases respectively [17]. NSCLCs are further subcategorised into adenocarcinoma, squamous cell carcinoma (SqCC) and large cell carcinoma [18]. Several FGFR aberrations have been described in the literature for NSCLC and in particular in SqCC which are summarised below (Table 1).

#### 1.2.1. Gene Amplifications

Among FGFR genetic abnormalities, *FGFR1* amplification has been reported to be the most common in NSCLC [15]. *FGFR* amplification has been shown to occur as a result of gene duplication or aberrant gene transcriptional control [33]. Amplification of the *FGFR* gene can lead to receptor overexpression at the cell membrane, which results in ligand-independent dimerisation by stochastic diffusion through the membrane. In a comprehensive study across 675 cases of NSCLC, SqCC showed the highest frequency of *FGFR1* amplification (~9%) compared to lung adenocarcinoma harbouring any FGFR abnormalities (~4%) [15]. Amplification of other FGFR family members is rare in lung cancer patients. A high frequency rate of *FGFR1* amplification in SqCC (13–22%) has also been reported in other independent studies [19,20,21,22,23]. Data from preclinical models of *FGFR1* amplified SqCC cell lines have shown that *FGFR1* amplification leads to protein overexpression and sensitivity to FGFR inhibitors, suggesting FGFR1 may represent an important therapeutic target in NSCLC [19,23]. Results from a recent study of 101 SqCC resected samples screened for *FGFR* amplification and mutations showed 22% of cases were positive for *FGFR1* amplification with only one patient showing an *FGFR3* mutation [24].

*FGFR1* amplification has also been reported in a small proportion of SCLC patients (~7%) [25,26,27]. Preclinical data have shown in vitro and in vivo activity of FGFR inhibitors on SCLC cell line growth [34]. More recently, a sustained response to the nonselective FGFR inhibitor pazopanib was reported in a heavily pretreated patient with *FGFR1*-amplified SCLC [35]. These results suggest that utilising FGFR antagonists might improve the outcome of patients with SCLC, a disease which has a very low survival rate and is traditionally considered refractory to targeted therapy [36].

#### 1.2.2. Point Mutations

Comprehensive analysis of genomic alterations in SqCC have found that mutations in *FGFR2* and *FGFR3* are present in ~3% of cases and account for the most frequent somatic mutations in this lung cancer subtype [15,28]. Mutations in the kinase domain are known to induce constitutive receptor activation, whereas mutations of the C-terminus tail can impair the autoregulatory processes of ubiquitination and internalisation [11,37]. The most frequent hotspot mutations identified in SqCC patients are the extracellular domain mutations W290C and S320C and the kinase domain mutations K660E and K660N in FGFR2. The most common mutations in FGFR3 are found in the kinase domain (R248C and S249C) [28]. Functional studies have established the oncogenic potential of these mutations in vitro and in mouse xenograft models [28]. Notably, when treated with a panel of FGFR inhibitors, NIH-3T3 and Ba/F3 model cell lines expressing FGFR2 W290C, and S320C mutants or FGFR3 R248C and S249C mutants, showed a significant reduction in cell survival, a reduction in cell transformation in anchorage-independent conditions and a reduction in tumour volume in xenograft mouse models [28]. Another study that sequenced 623 genes across 188 cases of lung adenocarcinoma identified four mutations in the kinase domain of FGFR4 [29]. One of these mutations (G681K) was previously identified in one lung adenocarcinoma specimen [38]. Interestingly, an analogous mutation was reported in glioblastoma in the Erb-B2 receptor tyrosine kinase gene (ERBB2) [39]. However, the biological significance of FGFR4 mutations in lung cancer remain to be determined experimentally.

#### 1.2.3. Chromosomal Translocations

A comprehensive genomic profiling analysis of 26,054 NSCLC patient specimens identified *FGFR* fusions in 0.2% (52/26,054) of cases with a higher frequency in SqCC (0.59%) than in adenocarcinoma (0.12%) [30]. This study focused on the analysis of *FGFR* fusions, where the *FGFR* kinase domain is retained and fused to an identifiable fusion partner. Notably, fusions with *FGFR* as the 5’ partner were more common than those with *FGFR* as the 3’ partner [30]. Three known and eleven novel FGFR fusion partners were identified, with the known activating fusion between *FGFR3* and transforming acidic coiled-coil containing protein 3 (*FGFR3*-*TACC3*) present in 71% of *FGFR* fusion positive cases (*n* = 37) [30]. The *TACC* gene encodes for a motor spindle protein dimer known to stabilise the mitotic spindle during cell division [40]. FGFR3-TACC3 proteins are usually in-frame fusions of the FGFR3 N-terminus with a TACC3 C-terminus. Due to the presence of a coiled-coil domain in *TACC3*, which enables TACC3 dimerisation, in *FGFR3*-*TACC3* fusions the dimerisation and activation of cellular signalling in the absence of ligand is elevated [41,42]. Because they are intracellular proteins and lack the C-terminus tail of FGFR3, these fusion proteins also often escape negative regulation mechanisms, impairing signal termination and protein degradation in the lysosomes [43,44]. Cells with this fusion protein are also particularly prone to aneuploidy due to the truncated form of TACC [42,45]. Other known fusions reported in this study were two involving *FGFR2* and shootin 1 (*FGFR2*-*SHTN1*), and another between BCL2 associated athanogene 4 and *FGFR1* (*BAG4*-*FGFR1*) [30]. *FGFR2*-*SHTN1* has been previously identified in cholangiocarcinoma and has been linked to increased phosphorylation of PI3K/AKT and the mechanistic target of rapamycin kinase (mTOR) pathways in in vitro assays in NIH-3T3 and 293T-engineered cells [46,47]. *BAG4*-FGFR1 has been previously reported in lung SqCC [31]. No functional studies on the mechanism of action of this fusion have been reported yet. Of the 52 total positive fusion cases, only four presented co-occurring known targetable driver alterations: two with epidermal growth factor receptor (*EGFR*) exon 19 deletions (Ex19del), and one with the EGFR L861Q point mutation and one with a *MET* exon 14 splice mutation [30]. Due to a lack of other known drivers, these fusions are likely to be the driving alteration in the remaining 48 patients. Another study, where fourteen known *FGFR* fusion variants were detected by RT-PCR and then verified by direct sequencing in 1,328 patients with NSCLC, found that *FGFR1* and *FGFR3* fusions occurred in 1.3% of NSCLC patients [31]. *FGFR3*-*TACC3* was confirmed as the most common translocation identified in fifteen lung cancer patients (1.1%), 6/1,1016 lung adenocarcinoma and 9/312 SqCC [31]. In addition, two cases of *BAG4*-*FGFR1* were found in SqCC, while no *FGFR2* fusions were identified in this study. The prevalence of *FGFR* fusions was higher among smokers (94.1%, 16/17 patients, *p* < 0.001) with SqCC in this study [31]. Finally, a single case of a fusion between *FGFR2* and citron Rho interacting kinase (*FGFR2*-*CIT*) was identified in a lung adenocarcinoma patient by the Cancer Genome Atlas project [32].

## 2. FGFR as a Mechanism of Resistance to Inhibition of EGFR and KRAS Mutations

In addition to aforementioned *FGFR* aberrations that are inherent in lung cancer, components of the FGFR pathway have also been shown to be altered in response to targeted therapy as a compensatory bypass mechanism to induce drug resistance. Here, we review FGFR pathway activation as a bypass mechanism to targeted therapies against *EGFR* and the Kirsten rat sarcoma 2 viral oncogene homolog (*KRAS*) mutations, the most common oncogenic drivers found in lung adenocarcinoma [48]. 

### 2.1. Resistance Associated with Use of EGFR Tyrosine Kinase Inhibitors

Activating mutations in *EGFR* are the second most prevalent oncogenic drivers present in ~15–20% of NSCLC cases [49,50]. The use of EGFR tyrosine kinase inhibitors (TKIs) in patients harbouring somatic EGFR-activating mutations is a poster child for targeted therapy, with significant improvement in the median progression-free survival (PFS) from 4.6 months to 13.1 months [51]. Despite initial response, acquired resistance almost inevitably occurs within ~16 months [52,53]. Over the past decade multiple mechanisms of resistance to EGFR inhibitors have been identified, including compensatory bypass signalling mediated by multiple RTKs [54,55]. The activation of the FGFR family of receptors has consistently been observed in preclinical models of EGFR TKI-resistant NSCLC [56,57,58,59]. A study from 2014 reported FGFR1 activation as a mechanism of resistance to the irreversible second-generation EGFR inhibitor afatinib in PC9 cells (a NSCLC cell line harbouring *EGFR* Ex19del) [56]. Afatinib-resistant subclones showed constitutive activation of FGFR through increased expression of FGFR1 and its ligand FGF2. Downstream signalling pathways, such as AKT and the extracellular signal-regulated kinases (ERK), were still highly phosphorylated in afatinib-resistant cells where expression of most of the EGFR family proteins was downregulated. In this study, no enhancement in expression of other FGFR family proteins were observed [56]. A similar result was reported by an independent study where EGFR TKI-resistant cell line models were established by long-term exposure to the competitive, reversible first-generation EGFR TKI gefitinib in PC9 and HCC827 (*EGFR* Ex19del) [57]. Gene expression analysis showed increased levels of FGFR1 and FGF2 in gefitinib-resistant cells. Notably, sensitivity to gefitinib in drug-resistant cells was restored by genetic silencing of either FGFR1 or FGF2 by siRNA, or by kinase inhibition using a nonselective, competitive FGFR inhibitor, PD173074 [57]. These findings were confirmed in a separate study utilizing several *EGFR* mutant NSCLC cell lines, which showed increased FGFR1 and FGF2 mRNA and protein levels in response to gefitinib exposure [59].

FGFR1 expression is often associated with epithelial to mesenchymal transition (EMT) [60,61]. EMT is responsible for loss of cell adhesion and increased invasion, migration and cellular proliferation. It occurs as a key step during physiological processes like embryonic morphogenesis, but also in the progression of primary tumours toward metastasis [62]. EMT has been linked to resistance to EGFR TKIs both in vitro and in NSCLC patients with activating EGFR mutations [63,64]. Azuma et al., showed an increased expression of EMT-related transcription factors, such as Snail and Twist, in afatinib-resistant cells [56]. Importantly, knockdown of these transcription factors impaired FGFR1 activation and overcame drug resistance [56]. A recent study where mesenchymal cell lines derived from biopsies of NSCLC patients who progressed on EGFR TKIs were analysed by whole-genome CRISPR screening, identified FGFR1 as the top target promoting survival of resistant cells to third-generation EGFR TKIs [61]. The authors proposed that combination therapy with EGFR and FGFR kinase inhibitors as a valuable strategy for resensitizing EGFR TKI-resistant cells that have undergone EMT [61].

The activation of an FGFR1/FGF2 autocrine loop is not the only FGFR-related mechanism of resistance that has been described. A previous study from Ware et al., reported increased FGFR2 and FGFR3 mRNA levels in a panel of NSCLC cell lines with *EGFR* activating mutations treated with gefitinib [58]. The authors demonstrated that gefitinib-induced FGFR2 and FGFR3 activation mediates ERK activation stimulated by FGF2 and FGF7 [58]. FGFR mutations were also identified as a key contributor to TKI resistance in a NSCLC patient-derived cell line [65]. A previously uncharacterized FGFR3 mutation, Y649C, located in the kinase domain, was found in the MGH156-1A cell line derived from a NSCLC patient who progressed on the first-generation, competitive EGFR TKI erlotinib, followed by a combination of afatinib with a monoclonal antibody (mAb) against EGFR, cetuximab. Importantly, FGFR inhibitors resensitized these cells to EGFR inhibitors, and the combination of FGFR with EGFR TKIs suppressed key signalling pathways such as AKT and ERK, suggesting that mutant FGFR3 could be an actionable therapeutic target [65]. Taken together, these data indicate that FGFR activation is a key bypass mechanism by which promotes the survival and growth of EGFR TKI-resistant cells [66].

### 2.2. Resistance Associated to Targeting Mutant KRAS in Lung Cancer

KRAS is another important oncogenic driver in lung cancer, with *KRAS* mutations accounting for approximately ~15–30% of NSCLC cases [67]. Therapeutic targeting of mutant *KRAS* lung cancers remains an unmet clinical need. In the past, most efforts have focused on the inhibition of downstream components of mutant *KRAS* driven pathways, such as MEK and CDK4/6 [68,69]. However, the initial efficacy of inhibitors such as the reversible and highly selective allosteric MEK1/2 inhibitor trametinib, or the reversible small molecule cyclin dependent kinase (CDK4/6) inhibitor palbociclib, is complicated by the rapid onset of acquired resistance [70,71,72]. 

FGFR1 was found to mediate acquired resistance to trametinib in a panel of *KRAS*-mutated lung cancer cell lines in a shRNA screen targeting the human kinome [72]. The authors showed that only shRNAs targeting FGFR1, but not other FGFR family members, conferred trametinib sensitivity [72]. As previously described, EMT has been found to confer acquired resistance to EGFR TKIs [73]. Kitai et al., reported that EMT can rewire RTK expression leading to differential feedback activation of the MAPK pathways following MEK inhibition in *KRAS*-mutant lung cancer cells [71]. Interestingly, resistance to MEK inhibitors is caused by the feedback activation of FGFR1-FRS2 pathway in mesenchymal-like *KRAS*-mutant lung cancer cell lines, NCI-H1792 and LU99. The combination of trametinib with the selective, ATP-competitive FGFR inhibitor infigratinib in these cells induced apoptosis in vitro, and tumour regression in xenograft mouse models. Notably, high *FGFR1* expression was also associated with mesenchymal-like *KRAS*-mutant adenocarcinoma patients [71].

Resistance to the CDK4/6 inhibitor palbociclib has been attributed to increased FGFR1 activity in the *KRAS*-mutant NSCLC cell line H358 [70]. Increased FGFR1 signalling was mediated by the upregulation and increased secretion of bFGF that, in turn, caused activation of an MEK-ERK-mTOR pathway. Consequently, FGFR1 inhibition with the pan-FGFR TKI LY2874455 led to palbociclib resensitization, suggesting the combinatorial inhibition of CDK4/6 with FGFR1 may be a promising strategy for the treatment of *KRAS*-mutant NSCLC [70].

Recently, small molecule compounds that covalently bind and modify the most common form of mutant *KRAS* in lung cancer, KRAS G12C, have shown promise in early clinical trials [74,75,76,77,78]. The activation of alternative RTKs or bypass signalling pathways have been associated with acquired resistance to KRAS G12C inhibitors in preclinical studies. Interestingly, Lito et al., showed that coinhibition of the KRAS G12C mutant (with KRAS inhibitor ARS-853) and FGFR (with PD173974), significantly reduced the proliferation of two lung KRAS G12C-mutant cell lines, H1792 and H2030 [79]. Another study reported increased phosphorylation levels of multiple RTKs, including FGFR, following 48 h treatment with the KRAS G12C inhibitor ARS-1620, but with high variability across different KRAC G12C-mutant cell line models, indicating cotargeting a single RTK is unlikely to be broadly effective [80]. However, the combination of ARS-1620 and FGFR selective inhibitor infigratinib displayed the greatest antiproliferative effect in a panel of KRAS G12C-mutant cells, including several lung cancer cell lines [80]. Finally, using a high-throughput drug screen, Misale et al., identified several RTK inhibitors, including the FGFR TKIs ponatinib and infigratinib, exhibiting strong synergies with ARS-1620 in NSCLC cells lines and patient-derived xenograft (PDX) models harbouring mutant KRAS G12C [81]. However, no obvious genetic event (FGFR amplification or mutations) were identified in these models [81]. Further preclinical and translational studies are required to determine the role of FGFR signalling in primary and acquired resistance to KRAS G12C targeted therapy.

Overall, aberrant activation of the FGF/FGFR signalling pathway makes a substantial contribution to targeted therapy resistance, promoting cellular proliferation, survival or EMT [82]. Targeting the FGFR pathways as a salvage therapy, or as part of an upfront combination, may therefore be useful in achieving more durable responses in patients with EGFR and KRAS mutations.

## 3. Preclinical and Clinical Studies of FGFR Inhibitors in Lung Cancer

Mutations and amplifications in *FGFR* are associated with poor survival in patients with NSCLC [21,83,84]. Therefore, there is an unmet clinical need for the development of targeted therapies directed against *FGFR* aberrations. To date, there are several therapeutic approaches to target FGFR family members involving selective and nonselective competitive FGFR TKIs. In this section we assess the current state of preclinical studies and clinical trials evaluating FGFR inhibitors for the treatment of lung cancers harbouring *FGFR* amplification, mutations and translocations. A list of FGFR agents that have been evaluated, or are currently undergoing clinical trials in NSCLC patients, is summarised in Table 2. 

### 3.1. Nonselective FGFR TKIs

ATP-competitive TKIs are small molecule inhibitors that interfere with the binding pocket of ATP in the tyrosine kinase domain of RTKs and, therefore, inhibit kinase activation and downstream signalling [95]. Due to high homology between kinase domains of different RTKs, a number of nonselective multitarget TKIs that inhibit FGFR in addition to a varied range of other RTKs, including vascular endothelial growth factor receptor (VEGFR), EGFR, platelet derived growth factor receptor (PDGFR), Fms related tyrosine kinase 3 (FLT3) and KIT, among others, have been developed [96]. Although this form of multitarget-based therapy has the potential to increase treatment efficacy by simultaneous blockade of redundant oncogenic pathways [66], it is often associated with increased patient toxicity and potential lack of bioactivity against the main oncogenic target of interest, therefore limiting their efficacy in tumours driven by aberrant FGFR signalling [97,98]. Moreover, as many of these multitarget RTKs also exert an inhibitory effect against angiogenic receptors such as VEGFR, it is difficult to deconvolute the effect of these drugs to the inhibition of FGFR alone [96,99]. Several TKIs have been evaluated in clinical trials in the context of NSCLC harbouring *FGFR* aberrations, or are still under investigation, and include ponatinib, dovitinib, lucitanib, cediranib, nintedanib and pazopanib [28,100]. Among them, ponatinib, dovitinib and pazopanib are the most clinically advanced.

Ponatinib (AP24534) is a multitarget kinase inhibitor approved for the treatment of chronic myelogenous leukemia (CML) and acute lymphoblastic leukaemia (ALL) [101]. Preclinical studies have shown antiproliferative activity of ponatinib in NSCLC cells expressing high FGFR1 levels [102,103,104]. Based on these preclinical data, a phase II trial (NCT01761747) was designed to study *FGFR1* amplification and mRNA expression as predictive markers for ponatinib sensitivity in NSCLC and SCLC patients. However, due to poor tolerability and safety concerns regarding ponatinib, the number of patients enrolled in the study that could be treated with ponatinib was low (*n* = 4) and the trial was terminated early without reaching its patient accrual target. Of the four patients, two showed stable disease (SD), with two patients progressed [85]. Another trial (NCT01935336) is currently ongoing to investigate the use of ponatinib in treating patients with advanced stage lung cancer. No results have been reported yet.

Dovitinib (TKI258) is a multitarget inhibitor that has shown modest efficacy and a tolerable safety profile in several cancers, including NSCLC [86]. In an open-label, single-arm phase II trial (NCT01861197), pretreated patients with advanced SqCC harbouring *FGFR1* amplification were treated with 500 mg dovitinib once daily. Data from 26 patients in this trial showed an overall response rate (ORR) of 11.5% after a median of 2.5 months treatment. The disease control rate was 50%, with three patients achieving partial response (PR), and the median progression-free survival (PFS) was 2.9 months. The most common grade 3–4 adverse events were fatigue, anorexia and hyponatremia [86].

Pazopanib (Votrient) is a multitarget drug currently approved for the treatment of metastatic renal cell cancer and soft-tissue sarcoma. In a case report study, a 49-year-old woman with heavily pretreated advanced SCLC carrying a *FGFR1* amplification showed a sustained response when treated with 800 mg pazopanib daily [35]. Interestingly, the *FGFR1* amplification was identified by analysing a liquid biopsy with an NGS plasma test and then confirmed by fluorescent in situ hybridization (FISH), demonstrating the utility of noninvasive monitoring for tumour genotyping in a disease that is classically associated with availability of small histological tumour specimens. 

While these inhibitors showed promising preclinical results, supporting the rationale to investigate their potential as therapies for lung cancer patients harbouring FGFR aberrations, toxicity is a major concern for this class of nonselective FGFR inhibitors. Clinical trials are often terminated due to severe adverse events, which provides a strong argument towards the need for more selective and effective first-line therapies for cancers with FGFR aberrations.

### 3.2. Selective FGFR TKIs

Selective FGFR inhibitors are a class of TKIs that have been specifically developed to exclusively target the FGFR family of receptors. Homology between the kinase domains of FGFR1/2/3 is higher compared to FGFR4 and, as a result, most of the TKIs in this class inhibit FGFR1-3 but not FGFR4 [105]. There are also some pan-FGFR inhibitors available that target all four isoforms of FGFR, as well as some selective FGFR4 inhibitors [106]. There is an array of different selective FGFR inhibitors that are currently being evaluated in clinical trials (Table 2). This includes the FGFR1-3 inhibitors AZD4547 and infigratinib, and the pan-FGFR inhibitors erdafitinib and rogaratinib.

AZD4547 is a potent, ATP-competitive FGFR1-3 inhibitor that can block FGFR signalling and reduce cellular growth in lung cancer cell lines and mouse xenograft models bearing *FGFR* alterations [107,108,109]. In a phase I clinical study (NCT00979134) within a cohort of 15 patients with previously treated stage IV, *FGFR1*-amplified SqCC, AZD4547 showed a PR for one patient with an 8% ORR. Two of 15 patients (13.3%) were progression-free at 12 weeks and the median PFS was 4.9 months. The most common related adverse events were gastrointestinal and dermatologic [88]. AZD4547 has also been investigated in the LUNG-MAP study (NCT02965378), a phase II clinical trial which includes a cohort of 27 pretreated patients with stage IV SqCC with a proven *FGFR* aberration, including *FGFR1/3* amplifications, FGFR3 S249C mutation and *FGFR3* fusions. Treatment with AZD4547 80 mg twice daily showed minimal activity, with only one out of 23 patients with *FGFR1* amplification achieving a PR which lasted less than three months, and one out of two patients with the FGFR3 S249C mutation having a PR for a duration 1.5 months [89]. Treatment with ADZ4547 had an acceptable safety profile with grade 3 adverse events occurring in six patients and grade 4 sepsis in one patient [89]. Despite minimal activity of AZD4547, the study has progressed into the phase III stage, where additional *FGFR* alterations are being investigated in recurrent or advanced SqCC.

Infigratinib (BGJ398) is a selective, ATP-competitive FGFR1-3 inhibitor discovered less than a decade ago. Preclinical studies have shown its potential as a selective FGFR inhibitor in the context of *FGFR2* and *FGFR3* point mutations in SqCC [28]. In cell line models expressing mutants FGFR2 W290C and S320C, or FGFR3 R248C and S249C, infigratinib was able to reduce the transformation properties of these cells in anchorage-independent conditions and reduce tumour volume in xenograft mouse models [28]. In a phase I/II clinical study (NCT01004224) involving patients with solid tumours harbouring FGFR alterations, infigratinib 125 mg/day was well tolerated and there was positive antitumour activity in 49 of 132 patients [92]. In the expansion arm evaluating 36 SqCC patients with *FGFR1* amplifications, four patients (~11%) achieved PR (three confirmed and one unconfirmed) and 14 patients had SD. The responders remained in the study for 39.9 to 76.6 weeks (confirmed PRs) and for 26.3 weeks (unconfirmed PR). Major adverse events were reversible hyperphosphatemia, stomatitis, alopecia, decreased appetite and fatigue [92]. Overall, these study results suggest a manageable safety profile and efficacy in *FGFR1*-amplified SqCC.

Erdafitinib (JNJ-42756493) is an ATP-competitive pan-FGFR inhibitor recently approved by the Food and Drug Administration (FDA) for the treatment of advanced or metastatic urothelial carcinomas with *FGFR3* and *FGFR2* alterations [110]. Preclinical data showed that erdafitinib has the potential to induce a prolonged inhibition of FGFR signalling, which is accompanied by reduced proliferation in a range of human lung cancer cell lines with *FGFR1* amplifications [111]. Perera et al., also showed that erdafitinib administration results in a potent and dose-dependent antitumour activity in xenograft models of the same cell lines [111]. Another study showed that erdafitinib and anti-PD-1 combination treatment induced changes in the tumour microenvironment to enhance antitumour response and survival in an *FGFR2*-mutant lung cancer mouse model [112]. Clinical data for erdafitinib has yet to be reported. However, two phase II clinical trials (NCT03827850 and NCT04083976) are ongoing in NSCLC patients with *FGFR* genetic alterations.

Rogaratinib (BAY1163877) is a selective, oral pan-FGFR inhibitor. Preclinical data has shown promising results in vitro and in vivo in several cell line and PDX models, including lung cancers with FGFR overexpression [113]. Several clinical studies have been conducted to investigate the antitumour activity of rogaratinib in solid tumours, including lung cancer with *FGFR* aberrations. The first-in-human study (NCT01976741) of rogaratinib to investigate the safety and maximum tolerated dose included a cohort of 36 patients with refractory advanced *FGFR* mRNA-overexpressing NSCLC. Rogaratinib administered at 800 mg twice daily was well tolerated with no dose-limiting toxicity and showed a 5.6% ORR (2 PR, 1 lasting > 16 months). Disease control rate was 64% [93]. A clinical trial is currently ongoing to investigate rogaratinib activity in *FGFR*-overexpressing patients with advanced and pretreated SqCC (NCT03762122).

CPL304110 is a selective, oral inhibitor of FGFR1-3. Preclinical data have showed a strong antiproliferative activity of this compound in a panel of human NSCLC cell lines harbouring *FGFR* aberrations and tumour growth inhibition in a gastric cancer *FGFR2*-amplified mouse xenograft model [94,114]. CPL404110 inhibited cell proliferation with a higher potency than AZD4547 [114]. Given these promising results, CPL304110 has recently entered a phase I clinical trial (NCT04149691) to assess its safety, tolerability and pharmacokinetics in advanced solid malignancies, including SqCC with *FGFR1-3* aberrations [94]. No results have been reported yet.

## 4. Mechanisms of Acquired Resistance to FGFR Inhibitors

**Figure 2:**
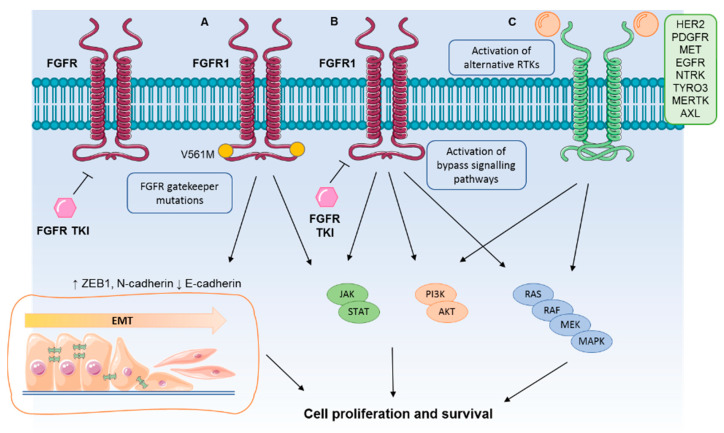
Mechanisms of acquired resistance to FGFR TKIs in lung cancer cell models. Preclinical evidence from the use of FGFR inhibitors in lung cancer models suggests drug resistance occurs through the (**A**) acquisition of gatekeeper mutations in FGFR and epithelial to mesenchymal transition, (**B**) activation of bypass signalling pathways such as JAK/STAT, PI3K/AKT and MAPK, and (**C**) activation of alternative RTKs.

Although selective inhibitors display a more favourable safety profile compared to nonselective FGFR TKIs, early clinical trials have shown that only a small fraction of lung cancer patients harbouring activating *FGFR* aberrations responded to FGFR inhibitors [13,86,88,92]. Future whole genome analyses may help define the exploitable molecular differences between responders and nonresponders, providing further insights into additional oncogenic drivers in NSCLC with *FGFR* aberrations. The drivers underpinning lack of response in some patients (intrinsic resistance) and disease progression on treatment (acquired resistance) with FGFR TKIs in lung cancer, are unknown. Mechanisms of FGFR TKI resistance are diverse and include the emergence of gatekeeper mutations, the activation of compensatory bypass signalling pathways, or alternative RTKs and induction of EMT (Figure 2). Although none of the above mechanisms have been observed in patients, understanding FGFR inhibitor resistance is an area of active research, and some of the mechanisms identified so far in preclinical studies in lung cancer models will be discussed here.

One of the most common TKI resistance mechanisms observed in patients with other oncogenic RTKs, such as EGFR or the anaplastic lymphoma receptor tyrosine kinase (ALK), is mutations in the gatekeeper residue of the kinase domain of the receptor [115]. Gatekeeper mutations are usually amino acid substitutions involving larger hydrophobic residues in the ATP-binding pocket site, which sterically obstructs drug access to the ATP-binding site or stabilises the active conformation of the kinase [116]. Several gatekeeper mutations in the FGFR family have been reported in preclinical studies in several cancer models, including FGFR1 V561M in lung cancer [117,118], FGFR2 V565I/N550K/V564F in endometrial cancer and cholangiocarcinoma [116,119], FGFR3 V555M in myeloma [115] and FGFR4 V550L/V550E in rhabdomyosarcoma [120]. Although FGFR gatekeeper mutations have not been identified in lung cancer patients yet, in vitro binding assays have shown that the gatekeeper residue V561M in FGFR1 is a mechanism of acquired resistance to FGFR inhibitors [23,117,118,121]. Kinetic analysis demonstrated that the FGFR1 V561M mutation confers a 38-fold increase in autophosphorylation of the receptor compared to wild-type. Interestingly, the mutated receptor exhibited a dramatic decrease in binding affinity to the nonselective FGFR inhibitor lucitanib, but only showed a modest decrease in affinity to AZD4547 [117]. However, cells expressing FGFR1 V561M are highly resistant to AZD4547 compared to wild-type, suggesting an alternative mechanism, other than the small reduction in binding affinity, may be responsible for driving V561M resistance to AZD4547 [118]. Analysis of the downstream signalling pathways in a *FGFR1*-amplified NSCLC cell line expressing the wild-type or V561M-mutated receptor, showed that resistance to AZD4547was mediated by the upregulation and high phosphorylation levels of STAT3 [118]. Furthermore, an increase in mesenchymal characteristics, together with increased proliferation, migration and invasion, was observed in these cells expressing V561M FGFR1 compared to wild-type, indicating that tumours that have acquired this resistance mutation may be more likely to metastasize to surrounding tissues [118].

Another known mechanism of resistance to selective kinase inhibitors is the activation of bypass signalling pathways that compensate for the blockade of FGFR-mediated survival signalling by activating alternative downstream effectors [122,123]. Due to the redundancy of intracellular signalling, and the cross-talk that exists between different pathways, cells can trigger adaptive responses to counteract their dependency on a particular signalling pathway (in this case FGFR signalling) to enable cell survival and tumour growth in the presence of kinase inhibitors [124]. A number of different compensatory mechanisms to selective FGFR inhibitors in the preclinical setting have been described in cell lines harbouring *FGFR1* amplification, such as the activation of the PI3K/AKT pathway upon FGFR inhibition with infigratinib in a SCLC cell line [125], and the activation of the JAK-STAT pathway in a NSCLC cell line chronically exposed to AZD4547 or infigratinib [126]. In addition, reprogramming of the cancer secretome to favour a cytokine-rich microenvironment has been shown to drive acquired resistance to FGFR TKIs through the activation of the transcription factor STAT3 in vitro and in vivo mouse lung xenograft models [126].

Another mechanism of compensatory signalling in response to kinase inhibitor therapy is the activation of alternative RTKs [54,55]. Coactivation of HER2 and PDGFRα caused by gene amplification or ligand overexpression has also been shown to activate the PI3K-AKT signalling and to reduce FGFR TKI-mediated MAPK signalling inhibition in an *FGFR1*-amplified lung cancer cell line [127,128]. Another study has shown increased MAPK pathway activation in both NSCLC and SCLC cell lines after long-term exposure to high doses of FGFR inhibitors such as AZD4547 and infigratinib [129]. Interestingly, different tumour models showed different mechanisms that cause subclonal emergence of resistance. The NSCLC drug-resistant cell line, H1581, presented an amplification on chromosome 1q12 that led to NRAS transcriptional upregulation, and deletion on chromosome 12p that resulted in significant downregulation of the dual specificity phosphatase 6 (DUSP6). In contrast, the SCLC drug-resistant cell line, DMS-114, exhibited transcriptional upregulation of MET. Accordingly, different combination treatments showed efficacy in resensitizing the two different resistant cell lines [129]. Genomic DNA quantitative PCR and FISH analysis also reported *MET* amplification in a AZD4547-resistant NSCLC cell line generated by long-term exposure to the drug [130]. Finally, upregulated MET expression at both the mRNA and protein level was also reported as a potential inducer of resistance to the FGFR1-3 inhibitor CPL304110 in a *FGFR1*-amplified NSCLC cell line subjected to chronic exposure to this compound [114]. Notably, inhibition of MET activity with capmatinib, a highly selective MET inhibitor recently approved by the FDA for the treatment of patients with metastatic NSCLC, restored sensitivity of this cell line to CPL304110 [114].

Results from a genome-wide functional screen to identify genes whose overexpression confers resistance to FGFR inhibitors in a *FGFR1*-amplified NSCLC cell line identified known resistance drivers such as MET, EGFR, AKT and MAPK, but also novel resistance mediators such as members of the neurotrophin receptor pathway (NTRKs) and the TAM family of tyrosine kinases (TYRO3, MERTK, AXL) [131]. In the same study, gene expression profiling of a large panel of resistant clones generated by chronic exposure to FGFR inhibitors in FGFR1- and FGFR3-dependant cell lines was also performed. Resistant clones showed increased mRNA levels in RAS, MAPK, the Erb-B receptor tyrosine kinase family (ERBB), PI3K and NTRK signalling pathways. Overall, these data suggest MAPK activation was the most common mechanism of resistance across models. Notably, infigratinib in combination with the reversible MEK inhibitor trametinib, was reported as the most successful approach in preventing FGFR resistance. However, the authors showed a diverse range of responses to FGFR inhibitors across different genomic background and lineages, highlighting the clinical challenge of rational combination therapy [131].

In summary, preclinical studies have shed light on some of the mechanisms of resistance to selective FGFR inhibitors [118,125,126,129,131]. However, as several bypass pathways and alternative RTKs may be activated in response to FGFR TKIs, it is difficult to predict the appropriate combinatorial strategy in each lung cancer patient harbouring *FGFR* aberrations. A better understanding of keys bypass signalling mechanisms that play a role in conferring resistance to FGFR TKIs is important to unveil new ways of effectively targeting compensatory survival pathways to attain long-term durable responses in patients with *FGFR* aberrations.

## 5. Strategies to Overcome FGFR TKIs Resistance

The emergence of multiple mechanisms to escape drug pressure presents a considerable clinical challenge for personalized therapy. In this section we will provide an overview of possible strategies to overcome FGFR TKIs resistance in lung cancer, focusing on new covalent investigational agents and targeted therapy combinations. 

### 5.1. Novel FGFR Therapies

Although FGFR gatekeeper mutations have not been observed in lung cancer patients yet, they are likely to be present at low frequency within patients with substantial intratumoural heterogeneity. The clinical experience gained in the field of EGFR mutant NSCLC have shown that the *EGFR* gatekeeper mutation T790M can be present at low abundance in tumours prior to treatment and be difficult to detect [132]. Furthermore, if patients are treated with inhibitors that are ineffective towards T790M, this may select for tumour cells that may be less responsive to subsequent therapies with third-generation EGFR inhibitors [133]. Anticipating such gatekeeper resistant mechanisms to first-generation FGFR inhibitors has led to the development of a second-generation of covalent FGFR inhibitors. Covalent inhibitors, such as the FGFR irreversible inhibitors 2 (FIIN-2) and 3 (FIIN-3), were designed to overcome clinical resistance by covalently binding to the ATP-binding pocket, potently inhibiting the proliferation of cells harbouring *FGFR* alterations including gatekeeper mutants [134,135]. However, both these covalent inhibitors proved to be unsuitable for in vivo treatment, as they showed only moderate mouse liver microsomal stability, which is used as a readout for the metabolic stability of the compound in vivo [136]. This issue required the development of an improved version FIIN-4, which showed improved mouse liver microsomal stability [136]. Although there are no active clinical trials evaluating these covalent inhibitors at the moment, they are of great interest for future therapies, including their use in the context of resistance to first-generation FGFR inhibitors.

### 5.2. Monoclonal Antibodies and FGF Traps

MAbs are also being investigated as an option to target FGFR or FGF ligands. Unlike TKIs, mAbs are designed to have a high affinity for antigens of selected FGFR isoforms or FGF ligands with less off target effects [12,137]. For example, antibody-based targeting of FGFR3 with R3Mab, an antagonistic anti-FGFR3 mAb capable of blocking ligand binding and receptor dimerisation, was tested in Ba/F3 mouse cells transfected with the cancer-associated mutants S249C, Y375C, R248C, G372C, and K652E [138]. R3Mab was able to inhibit the activation of mutant FGFR3s and the immediate downstream signalling pathway MAPK. Ligand-independent proliferation was suppressed on all cysteine mutants, while ligand-induced proliferation was abolished for the K652E mutant [138]. 

Aprutumab ixadotin (BAY 1187982) is a novel antibody-drug conjugate (ADC) that has shown preclinical efficacy against *FGFR2*-mutant cancer cell lines [139]. It consists of a human FGFR2 mAb conjugated through a noncleavable linker to a novel derivative of the microtubule-disruptive cytotoxic drug auristatin. ADCs represent a promising therapeutic approach for cancer treatment, as they combine the specificity of a mAb with the targeted delivery of a highly potent cytotoxic drug [140]. However, toxicity may be an issue with this particular antibody as an open-label, multicenter, phase I dose-escalation trial (NCT02368951) in patients with advanced solid tumours had to be terminated due to severe dose-limiting toxicities.

Another alternative therapeutic approach is the use of FGF traps, a structurally heterogeneous group of molecules able to bind and sequester FGF ligands, thus preventing their interaction with FGFRs [141]. An example is FP-1039 (GSK3052230), which consists of the extracellular domain of FGFR1 fused to the fragment crystallizable (Fc) region of immunoglobulin G1 (IgG1) [142]. FP-1039 selectively binds and neutralizes several FGFs that normally bind FGFR1, blocking the interaction between FGF-FGFR1 and, therefore, preventing receptor activation. It is being evaluated in combination with paclitaxel and carboplatin, or docetaxel, or as single agent in a multiarm, multicenter, open-label phase Ib study (NCT01868022) in metastatic SqCC. The clinical trial is ongoing and no data have been reported yet.

### 5.3. Combinatorial Strategies

As more effective drugs are developed to overcome secondary resistance mutations in the targeted genes, the activation of compensatory signalling pathways of resistance will likely continue to emerge in the clinical setting. In this regard, therapeutic success can be achieved when combinations of inhibitors that result in rapid and synergistic suppression of coactivated pathways are used, thereby preventing the emergence of drug resistance [143]. As extensively discussed in the previous section, preclinical studies have shown promising results when combinatorial targeted therapies are used to impair the development of acquired resistance to selective FGFR inhibitors in *FGFR*-aberrant lung cancer cells and mouse models [125,127,130,131,144]. Examples of effective TKIs combinations in preclinical lung cancer studies are the coinhibition of FGFR and MEK or MET signalling pathways. This has been achieved using infigratinib, which targets FGFR, and either trametinib, which targets MEK, or crizotinib and capmatinib, which target MET [114,129,131]. In addition, another study from 2015 identified mTOR as the most relevant synthetic lethal mediator that contributes to ponatinib intrinsic sensitivity in a panel of *FGFR1*-amplified NSCLC cell lines. Combinatorial treatment with mTOR inhibitors, AZD8055 or AZD2014, and FGFR TKIs, such as AZD4547 and ponatinib, was found to inhibit tumour growth in these cell lines [103]. Such combination approaches could represent a rationale treatment strategy to prevent the acquisition of drug resistance in lung cancers carrying *FGFR* aberrations.

A lesson learned from NSCLC patients with activating mutations in oncogenic drivers, such as EGFR, ALK and BRAF, underlines that treatment regimens which utilise sequential drugs with different mechanisms of action to target drug-sensitive clones, without allowing proliferation of drug-resistant cells, may be an effective strategy to minimize the evolution of drug resistance [145]. Recently, a novel computational framework was used to integrate experimental data and model tumour evolutionary dynamics to generate optimal drug scheduling strategies in real-time [146]. In these simulations, a drug switching strategy was superior to static drug combinations to restrict drug resistance and control overall tumour growth for longer periods of time. Furthermore, computational modelling and in vitro experimental data supported a hypothesis of ‘temporal collateral sensitivity’ in cancer cells [147,148]; where genetic variations present in tumours evolving in response to drug A may render at least a proportion of the tumour more susceptible to a different drug B, and that this enhanced treatment efficacy may occur within a restricted time-window following initial therapy. It would be interesting to evaluate if sequential drug schedules of FGFR and other inhibitors that target compensatory signalling pathways, are superior to combination therapies. 

## 6. Future Perspectives

To date, FGFR-targeted monotherapy has shown a lower response rate in NSCLC patients harbouring *FGFR* aberrations than expected based on preclinical data, suggesting that *FGFR1* amplification may not be a robust predictor of response to FGFR TKI inhibition [89,92,93]. These results may partly be explained by the presence of heterogeneous amplicons around the 8p11 genomic region leading to false positive results when *FGFR1* amplification is assessed by FISH rather than by NGS [15,149]. Moreover, it has been reported that both gene amplification and increased protein expression are required for patient stratification when predicting FGFR TKI sensitivity in *FGFR1*-amplified lung cancer [104,127]. Further investigation to find additional biomarkers, which may be better able to predict response to FGFR inhibitors in the clinical setting, remains of paramount importance. 

PDX models of NSCLC have been used to identify biomarkers of response to FGFR inhibitors. For instance, PDX models have been used to identify biomarkers to predict treatment response to AZD4547 of *FGFR1*- and *FGFR4*-overexpressing NSCLC tumours [108]. This study, performed on five different NSCLC PDX models with low and high N-cadherin expression, demonstrated that FGFR1 and FGFR4 overexpression alone had no prognostic potential, while coexpression of FGFR1 and FGFR4 with N-cadherin may predict for AZD4547 treatment efficacy [108]. These findings may, in part, explain the modest efficacy of FGFR TKIs in the clinic. However, further work is required to validate these data and establish the cut-off expression levels of these proteins for predicting FGFR inhibitor efficacy. 

MicroRNAs (miRNAs) have also been utilised as biomarkers to predict sensitivity to ponatinib and AZD4547 in a panel of histologically diverse lung cancer cell lines [150]. Among the 34 cell lines tested, 14 showed ponatinib sensitivity and 20 exhibited AZD4547 sensitivity (IC_50_ < 100 nmol/L). Comprehensive analysis of miRNA expression identified a miRNA signature, including let-7c, miRNA155 and miRNA218, that predicts for FGFR TKI response. In addition, the authors reported that let-7c may be involved in regulating FGFR1 expression as let-7c silencing was significantly associated with decreased FGFR1 mRNA levels [150]. Further studies on tumour tissue specimens from patients would be required to validate the potential role of this miRNA signature as a biomarker for predicting FGFR TKIs sensitivity. Importantly, analysis of circulating miRNA from liquid biopsies was identified as a reliable, less invasive tool for screening patients with *FGFR1*-overexpressing tumours and could be used for the validation of this miRNA signature.

## 7. Conclusions 

The incidence of *FGFR* aberrations as oncogenic drivers in lung tumours has prompted the search for new, more potent, selective FGFR inhibitors. Preclinical studies and early phase trials have demonstrated that there are a diverse range of responses to FGFR TKIs therapy across lung cancer patients harbouring FGFR aberrations, and that resistance occurs to these targeted agents [151]. Preclinical findings have reported several candidate resistance mechanisms to FGFR inhibition, including gatekeeper mutations and the activation of compensatory mechanisms that evade oncogene dependency. While cancer genome sequencing data have provided detailed information about the distribution and frequencies of *FGFR* alterations in different cancer types, and structural studies have elucidated the consequences of some of these mutations at the molecular level, the functional mechanisms by which these mutations promote cell survival have yet to be fully described. Similar to other oncogenic RTKs, like EGFR and ALK, distinct FGFR mutants might harbour different signalling dependencies, driving cancer progression by the activation of altered downstream pathways. Several challenges remain which hinder the effective use of selective FGFR kinase inhibitors in the clinic. We do not fully understand why some mutations fare better than others when treated with these drugs, which requires a deeper analysis of the biochemical and oncogenic properties of different FGFR mutations. There is also a need for the development of robust biomarkers for patient stratification to FGFR inhibitor therapy to enable better patient selection in order to improve the clinical effectiveness of these targeted therapies. 

## Figures and Tables

**Table 1:** Common FGFR genomic alterations found in lung cancer.

Gene	Alteration	Histology	Incidence (%)	Ref.
*FGFR1*	Amplification	NSCLC SqCC	6–22	[15,19,20,21,22,23,24]
*FGFR1*	Amplification	SCLC	7	[25,26,27]
*FGFR2*	Somatic mutations; W290C, S320C, K660E/N	NSCLC SqCC	3	[15,28]
*FGFR3*	Somatic mutations; R248C, S249C	NSCLC SqCC	3	[15,28]
*FGFR4*	Somatic mutations; G2041A	NSCLC adenocarcinoma	2	[29]
*FGFR3*	Translocations; *FGFR3-TACC3*	NSCLC, prevalently SqCC	0.1–1.1%	[30,31]
*FGFR2*	Translocations; *FGFR2-SHTN1, FGFR2-CIT*	NSCLC adenocarcinoma	rare	[30,32]
*FGFR1*	Translocations *BAG4-FGFR1*	NSCLC SqCC	rare	[30,31]

**Table 2:** Key clinical trials evaluating lung cancer patients harbouring FGFR alterations. Details for trials with NCT numbers can be accessed on https://clinicaltrials.gov (accessed on 2 April 2021).

Inhibitor (Manufacturer)	Target	Clinical Trial Identifier	Patient Characteristics	Regimen	Phase Study	Status/Ref.
**Nonselective inhibitors**						
Ponatinib (ARIAD, Pharmaceuticals)	FGFR, PDGFR, VEGFR, ABL, SRC, KIT	NCT01761747	Advanced NSCLC; *FGFR1* alterations	Ponatinib monotherapy	II	Terminated [85]
		NCT01935336	Advanced lung cancer, all histologies; *FGFR* SISH/ISH ^1^	Ponatinib monotherapy	II	Active, not recruiting [85]
Dovitinib (Allarity Therapeutics)	FGFR1-3, VEGFR1-3, PDGFRβ, FLT3, KIT, RET, TRKA, CSF1	NCT01861197	Advanced SqCC; *FGFR1* amplification	Dovitinib monotherapy	II	Unknown [86]
Pazopanib (Novartis	FGFR1-3, VEGFR1-3, PDGFR, KIT	Case report study	Advanced SCLC; *FGFR1* amplification	Pazopanib monotherapy		[35]
Nintedanib (Boehringer-ingelheim)	FGFR1-4, VEGFR1-3, PDGFRα-β	NCT01948141	Advanced SqCC; *FGFR1* amplification	Nintedanib monotherapy	II	Completed
Lucitanib (HaiHe Biopharma)	FGFR1, VEGFR1-3	NCT01283945	Advanced NSCLC; *FGFR1* amplification	E3810 monotherapy	I/II	Completed [87]
**Selective inhibitors**						
AZD4547 (AstraZeneca)	FGFR1-3	NCT00979134	Advanced SqCC; *FGFR1* amplification	AZD4547 monotherapy	I	Terminated [88]
		NCT02965378	Advanced SqCC; *FGFR* alterations	AZD4547, docetaxel	II/III	Active, not recruiting [89]
		NCT01824901	Advanced SqCC; *FGFR1* amplification	Docetaxel with or without AZD4547	I/II	Completed [90]
		NCT01795768	Advanced SqCC; *FGFR1*-or *FGFR2*-amplified tumours	AZD4547 monotherapy	II	Unknown [91]
		NCT02154490	Advanced SqCC; FGFR1-3 positive tumours	AZD4547, docetaxel	II/III	Active, not recruiting
Infigratinib (QED Therapeutics)	FGFR1-3	NCT01004224	Advanced SqCC; *FGFR1* amplification	Infigratinib monotherapy	I/II	Completed [92]
Erdafitinib (Janssen Pharmaceuticals)	FGFR1-4	NCT03827850	Advanced NSCLC; *FGFR* alterations	Erdafitinib monotherapy	II	Recruiting
		NCT04083976	Advanced NSCLC; *FGFR* alterations	Erdafitinib monotherapy	II	Recruiting
Rogaratinib (Bayer)	FGFR1-4	NCT01976741	Advanced NSCLC; *FGFR* alterations	Rogaratinib monotherapy	I	Completed [93]
		NCT03762122	Advanced SqCC; FGFR mRNA overexpression	Rogaratinib monotherapy	II	Active, not recruiting
CPL304110 (Celon Pharma)	FGFR1-3	NCT04149691	Advanced SqCC; *FGFR1-3* alterations	CPL304110 monotherapy	I	Recruiting [94]

^1^ SISH, silver in situ hybridization; ISH, in situ hybridization.
